# Cardiovascular Disease Burden in Rural Central Asia: A Systematic Review of Epidemiological Trends and Mortality Patterns

**DOI:** 10.3390/epidemiologia7010010

**Published:** 2026-01-06

**Authors:** Akerke Kassymkhan, Alma-Gul Ryskulova, Zhanara Buribayeva, Bakytgul Nurmukhambetova, Kenzhebek Bizhanov, Daria Nabok, Nargiza Nassyrova, Magripa Bapayeva, Erkin Mirrakhimov

**Affiliations:** 1 Department of Public Health and Social Sciences, Department of Internal Medicine, Kazakhstan’s Medical University “KSPH”, Almaty 050060, Kazakhstan; r.alma@bk.ru (A.-G.R.); m_mother@mail.ru (M.B.); 2 Department of Internal Medicine, Department of Surgery, Faculty of Medicine and Healthcare, Al-Farabi Kazakh National University, Almaty 050040, Kazakhstan; kenzhebek.bizhanov07@gmail.com; 3 Department of Nursing, Department of Neurology, Department of Internal Medicine, Asfendiyarov Kazakh National Medical University, Almaty 050012, Kazakhstan; mm-antai@mail.ru (Z.B.); nurmuhambetova.b@kaznmu.kz (B.N.); 4 Department of Interventional Cardiology, Arrhythmology and Endovascular Surgery, Syzganov National Scientific Center of Surgery, Almaty 050004, Kazakhstan; 5 Department of Cardiology, Karaganda Medical University, Karaganda 100009, Kazakhstan; leontyevna.d.n@gmail.com; 6 Department of Research, Kazakh-Russian Medical University, Almaty 050004, Kazakhstan; nassyrova.n94@gmail.com; 7 Department of Faculty Therapy, Kyrgyz State Medical Academy Named After I.K. Akhunbaev, Bishkek 720020, Kyrgyzstan; erkmirr@gmail.com; 8 College of Medicine, Korea University, Seoul 02841, Republic of Korea

**Keywords:** cardiovascular diseases, urban–rural disparities, epidemiology, incidence, prevalence, mortality, modifiable risk factors, Central Asia, public health

## Abstract

**Background/Objectives:** Cardiovascular diseases (CVDs) remain a leading cause of mortality worldwide, with a particularly high burden in Central Asian countries. Despite ongoing urbanization, rural populations constitute a significant demographic in this region, yet epidemiological data stratified by urban and rural residence are limited and fragmented. This systematic review aimed to synthesize current evidence on the incidence, prevalence, mortality, and risk factor profiles of CVDs among urban and rural populations in Central Asia, identify disparities, and inform targeted prevention and control strategies. **Methods:** A systematic literature search was conducted across the PubMed, Science Direct, Web of Science, and Google Scholar databases for studies published between 2015 and 2025. Included studies reported cardiovascular health indicators with urban–rural stratification in Kazakhstan, Kyrgyzstan, Uzbekistan, Tajikistan, and Turkmenistan. Data extraction and qualitative synthesis were performed, with methodological quality assessed using the Newcastle–Ottawa Scale. **Results:** Eight original studies met the inclusion criteria, encompassing national and regional datasets with diverse designs, including retrospective analyses, cross-sectional surveys, and registry data. Overall, CVD incidence and prevalence showed increasing trends in both urban and rural areas, with consistently higher mortality rates in urban populations. Key modifiable risk factors—hypertension, obesity, dyslipidemia, and smoking—were prevalent, particularly in rural settings. Variability in healthcare access and preventive program implementation contributed to the observed disparities. Limited data from some countries, particularly Tajikistan and Turkmenistan, highlight gaps in epidemiological surveillance. **Conclusions:** The cardiovascular disease burden in Central Asia demonstrates significant urban–rural disparities, underscoring the need for tailored public health interventions and enhanced healthcare resource allocation in rural regions. Strengthening epidemiological monitoring and implementing region-specific prevention programs targeting modifiable risk factors are imperative for reducing CVD morbidity and mortality. Further high-quality research is necessary to address existing data gaps and optimize cardiovascular health strategies across the region.

## 1. Introduction

Cardiovascular diseases occupy a leading position among non-communicable diseases, determining the bulk of the global burden of disease and being the leading cause of death worldwide [1,2]. In 2019, out of 56.5 million deaths, 18.6 million (32.9%) were due to CVDs [3]. The importance of monitoring them is associated not only with medical but also with international obligations, including the United Nations Sustainable Development Goals, which call for a reduction in premature mortality from non-communicable diseases (NCDs) by a third, including by reducing mortality from ischemic heart disease [4]. In 2019, the global burden of coronary heart disease (CHD) was 197.2 million (95% UI: 177.7–219.5) cases, 9.1 million (95% UI: 8.4–9.7) deaths, and 182.0 million (95% UI: 170.2–193.5) disability-adjusted life years (DALYs), with age-standardized rates declining by 4.6%, 30.8%, and 28.6%, respectively, since 1990 [5]. In 2021, they caused 20.5 million deaths, about a third of all deaths, with more than 75% of these deaths occurring in low- and middle-income countries [6]. Central Asia remains one of the world leaders in the burden of CHD: in 2019, the prevalence reached 2,763,285 cases (95% UI: 2,544,108–3,006,299) with an age-standardized rate of 4131.8 per 100,000 (95% UI: 3827.6–4475.2), which is 2.3% higher than in 1990; mortality was 200,135 cases (95% UI: 183,535–218,271) with a rate of 360.0 per 100,000 (95% UI: 330.9–390.2) [7]. The above data show that Central Asia, including Kazakhstan, Kyrgyzstan, Uzbekistan, Tajikistan, and Turkmenistan, is among the regions with a high burden of cardiovascular diseases and comparatively high mortality rates from diseases of the circulatory system [8,9].

A significant feature of this macroregion is the predominance of the rural population: in several countries, the proportion of rural residents exceeds 50%, and in some states, it exceeds two-thirds [10,11,12]. Despite the high prevalence of CVDs in this region, data on morbidity and mortality in rural areas remain fragmented, often presented only in a generalized form without being separated into urban and rural indicators [13,14,15]. As is known, in many countries, the organization of healthcare in cities and rural areas differs significantly [16,17,18]. Urban centers are characterized by higher accessibility to specialized medical care, modern diagnostic equipment, and qualified personnel, while rural areas often face a shortage of personnel, limited material and technical base, and geographical remoteness from high-level medical institutions [19,20]. These differences create significant barriers to the timely diagnosis and treatment of CVDs, which can contribute to worse outcomes in rural residents [21].

This information gap hinders the development of targeted prevention and treatment strategies in vulnerable populations. This systematic review aims to summarize the available epidemiological data on CVD morbidity and mortality in rural areas of Central Asian countries to identify the specificity and scale of the problem.

## 2. Materials and Methods

The study protocol was registered in the PROSPERO International Prospective Register of Systematic Reviews of the National Institute for Health Research (ID: CRD420251164147) [22].

### 2.1. Search Strategy

**Figure 1:**
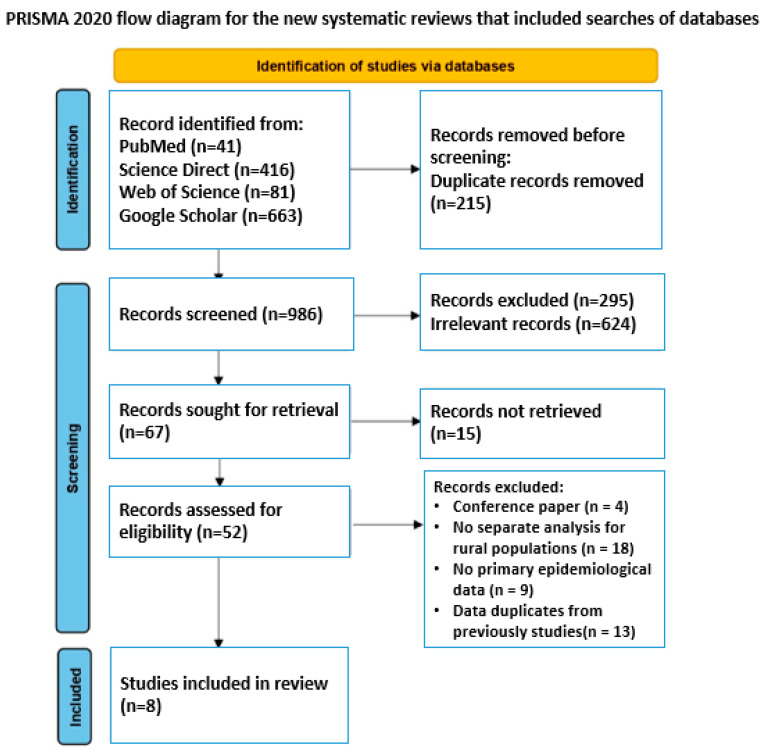
Flow chart of the PRISMA study selection process.

A preliminary search of the PROSPERO database did not identify any registered protocols addressing cardiovascular diseases in rural populations of Central Asia. A systematic literature search was conducted in accordance with the PRISMA (Preferred Reporting Items for Systematic Reviews and Meta-Analyses) guidelines [23] to ensure transparency and reproducibility of the analytical process, as detailed in Figure 1. The completed PRISMA checklist (Table S1) is provided in the Supplementary Materials.

The search covered publications from 1 January 2015 to 1 June 2025, and was performed in five international bibliographic databases: PubMed, Science Direct, Google Scholar (English and Russian interfaces), and Web of Science. The search strategies combined controlled vocabulary (MeSH terms) and free-text keywords related to “cardiovascular disease”, “mortality”, “morbidity”, “epidemiology”, “rural”, “urban”, and “Central Asia”, as well as the names of individual countries (Kazakhstan, Kyrgyzstan, Uzbekistan, Tajikistan, Turkmenistan) in both English and Russian. Boolean operators were applied to refine the queries, and syntax was adapted to the requirements of each database. The search strategies for each database are summarized in the Supplementary Materials below (Table S2).

The initial search yielded 1201 records. After removing 215 duplicates, 986 unique titles and abstracts were screened. Based on the inclusion and exclusion criteria, 934 publications were excluded at the title and abstract screening stage (e.g., studies outside Central Asia, absence of rural–urban stratification, non-CVD focus, or insufficient epidemiological data). Full-text assessment was performed for 52 articles, of which 44 were excluded due to certain reasons. As a result, 8 original studies met all inclusion criteria and were included in the final systematic review.

Despite the existence of a limited number of cardiovascular disease studies from Turkmenistan and Tajikistan, none were identified that specifically focused on rural populations. As a complementary component to this review, and to provide a more comprehensive epidemiological overview, we additionally examined publicly available official statistics on the incidence, prevalence, and mortality of cardiovascular diseases from the national statistical offices of all Central Asian countries.

### 2.2. Eligibility Criteria

This systematic review included original epidemiological studies conducted in Central Asian countries (Kazakhstan, Kyrgyzstan, Uzbekistan, Tajikistan, and Turkmenistan) that reported data on the incidence, prevalence, mortality, or disability-adjusted life years from cardiovascular diseases with stratification by rural and urban populations or exclusively for rural populations. We considered studies based on national statistics, population surveys, and cohort analyses, regardless of whether the data covered the entire population or specific age groups.

Definition of cardiovascular disease

For the purposes of this review, we applied the expanded ICD-10 classification of cardiovascular diseases (I00–I99), which is used in official national reporting systems of Kazakhstan, Kyrgyzstan, Uzbekistan, Tajikistan, and Turkmenistan. This definition includes ischemic heart disease, cerebrovascular disease, hypertension, heart failure, rheumatic heart disease, and other circulatory disorders. In contrast, the Western literature often employs a narrower concept of “major CVD”, limited to ischemic heart disease and stroke. Given the structure of available regional data, the full ICD-10 definition was used to ensure consistency and comparability across studies.

Studies were excluded if they did not contain stratified data by place of residence, lacked cardiovascular disease outcomes, or were review articles, case reports, letters, posters, dissertations, conference abstracts, or other forms of grey literature, as well as publications presenting duplicate datasets without new information (Table 1).

### 2.3. Selection of Studies and Data Extraction

After removing duplicate records, the identified publications underwent step-by-step screening by two independent reviewers. In the first stage, titles and abstracts were assessed to determine relevance to the study topic. Articles that met the inclusion criteria proceeded to full-text review. The final decision on inclusion was made after a thorough comparison of the article’s content with the predefined eligibility criteria.

Data extraction was also conducted independently by two researchers. In cases of disagreement in interpretation or coding, discussions were held, and a third expert was involved when necessary to reach a consensus. The following information was recorded for each included study: first author’s name, year of publication, country and specific region of the study, study design, data collection years, sample size, age groups (if reported), urban–rural classification (if available), main cardiovascular disease indicators (incidence, prevalence, mortality, DALY), values of these indicators, and the data collection methods used. When information was incomplete or missing, details were retrieved directly from the text, Supplementary Materials, or tables. All extracted data were verified, standardized, and organized into a unified database for subsequent analysis.

### 2.4. Risk of Bias (Quality) Assessment

To evaluate the methodological quality of the included studies, we applied the Newcastle–Ottawa Scale (NOS) in its version for cohort and retrospective comparative research, following the recommendations of the Cochrane Collaboration Working Group for the appraisal of non-randomized studies [24]. The NOS assesses three main domains: participant selection (maximum 4 points), comparability of study groups (maximum 2 points), and outcome assessment (maximum 3 points). The total score ranges between 0 and 9, where higher scores indicate stronger methodological rigor and a lower risk of bias.

Quality assessment was conducted independently by two reviewers. Any differences in scoring were resolved through discussion, and, when necessary, a third reviewer was consulted to reach an agreement. Studies scoring ≥7 points were considered as high quality, those with 5–6 points as moderate quality, and those with ≤4 points as low quality. The quality ratings were documented and considered during the synthesis of the findings.

No studies were excluded solely based on quality score. However, for research with a higher risk of bias, their contribution to the final conclusions was interpreted cautiously, with attention to study design limitations and potential confounders. A summary of the NOS scores for all included studies is presented in Table 2.

Due to the limited number of eligible studies (*n* = 8) and substantial variability in study designs, outcome measures, reference periods, and data sources, a quantitative meta-analysis was deemed inappropriate. The heterogeneity in the included studies—in terms of methodology, regional focus, and completeness of rural–urban stratification—precluded meaningful statistical pooling. Therefore, the synthesis was performed qualitatively, with careful consideration of the context, strengths, and limitations of each study.

## 3. Results

### 3.1. Demographic Profile and Cardiovascular Mortality in Central Asian Countries

As of January 2025, the combined population of the five Central Asian countries—Kazakhstan, Uzbekistan, Kyrgyzstan, Tajikistan, and Turkmenistan—amounted to more than 83 million people. Despite ongoing urbanization processes, rural populations still represent a substantial proportion in most countries of the region, with particularly high shares in Tajikistan and Kyrgyzstan. The distribution of total, urban, and rural populations for each country is summarized in Table 3.

Analysis of official national statistical reports reveals considerable variation in mortality from diseases of the circulatory system (International Classification of Diseases (ICD)-10 codes I00-I99) across Central Asian countries in recent years. In Uzbekistan, publicly available statistics indicated relatively high and stable CVD mortality over the period 2020–2023, while Turkmenistan reported the highest national figure in the region (552 per 100,000 in 2021). Among the five Central Asian countries, Kazakhstan and Tajikistan were the only states that provided rural–urban disaggregated cardiovascular mortality data. For Kyrgyzstan, Uzbekistan, and Turkmenistan, only national-level mortality statistics were available, which limits the feasibility of a full cross-country rural–urban comparison. Where disaggregated data were not available, gaps are noted in Table 4.

**Figure 2:**
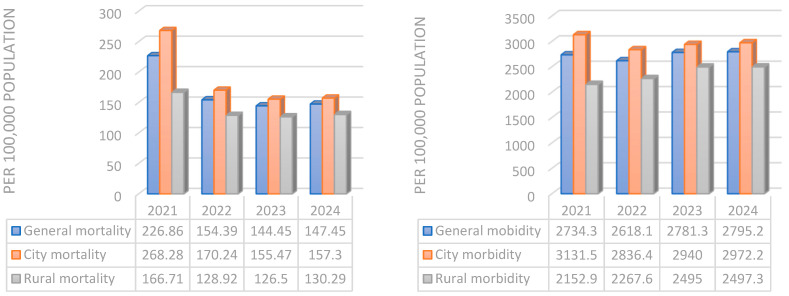
CVD mortality and morbidity in Kazakhstan’s city and rural populations.

Kazakhstan showed a steady decline in overall CVD mortality between 2021 and 2023, with a slight increase in 2024. The available urban–rural breakdowns consistently demonstrate higher mortality rates in urban areas compared with rural populations (Figure 2).

In Kyrgyzstan, although urban–rural disaggregated data were not publicly available, the national mortality rate decreased from 317.7 per 100,000 in 2020 to 230.3 per 100,000 in 2024. Tajikistan also demonstrated a downward trend, with urban mortality rates exceeding rural values in all reported years.

### 3.2. Included Study Characteristics

The final synthesis comprised eight original studies [25,26,27,28,29,30,31,32] that fully met the predefined inclusion criteria. All were published between 2020 and 2024 and employed either retrospective analyses of official health statistics [25,26,28,29,31] or epidemiological study designs, including cross-sectional surveys and registry-based descriptive research [27,28,31,33]. Most investigations were regional or national in scale, with several providing a breakdown by urban and rural populations [25,26,27,28,29,31], while others focused exclusively on rural communities [27,30,32]. Sample sizes varied considerably, ranging from 1330 participants in targeted rural epidemiological surveys [30] to national datasets covering millions of individuals [25,26].

The included studies reported a wide range of cardiovascular health indicators, including incidence, prevalence, and mortality for overall cardiovascular disease, ischemic heart disease (IHD), acute myocardial infarction (AMI), cerebrovascular disease (CeVD), and arterial hypertension (AH) [25,26,27,28,29,30,31]. Risk factor assessments included smoking, obesity, dyslipidemia, and other major modifiable determinants of cardiovascular health [27,30,32]. Data collection methods varied and included analyses of national health databases [25,26,28,29,31], outpatient registry reviews [30], structured questionnaires [27,30,32], physical examinations [27,28,29,30,31,32], laboratory testing [27,32], and electrocardiography [30].

It is worth noting that while several studies provided longitudinal or comparative data across urban and rural settings [25,26,27,29,30], no eligible publications were found that analyzed rural CVD epidemiology in Turkmenistan or Tajikistan. For a broader contextual understanding, national-level statistics from these countries were reviewed separately (Table 5). A detailed summary of the characteristics of the included studies is presented in Table 5.

The nine included studies demonstrated considerable variability in cardiovascular disease burden and risk factor profiles across Central Asian countries. In Kazakhstan, several national and regional analyses reported rising trends in arterial hypertension and increasing cardiovascular incidence, while mortality from ischemic heart disease and cerebrovascular disease showed a steady decline in recent years [25,26,27,28,29,30]. Urban–rural differences were consistently observed, with urban areas generally demonstrating higher incidence and mortality, although certain rural regions exhibited particularly elevated burdens.

Evidence from Kyrgyzstan revealed substantial prevalence of major modifiable risk factors—including hypertension, obesity, dyslipidemia, and smoking—across rural populations [29,30]. A retrospective analysis from the Osh region further demonstrated high rural incidence and mortality, though values were lower than in urban Osh [31]. In Uzbekistan, large-scale screening data highlighted high rates of smoking and alcohol use, with obesity affecting nearly 10% of rural adults [32]. Given the diversity of study designs, outcome measures, and population characteristics, the key findings from each investigation are synthesized in Table 6.

The key trends and main findings identified in the selected studies are summarized in Table 7.

## 4. Discussion

This systematic review integrates epidemiological evidence and healthcare disparities related to cardiovascular disease in Central Asia, focusing on rural–urban differences across Kazakhstan, Kyrgyzstan, and Uzbekistan.

The demographic profile of Central Asian countries, characterized by a substantial proportion of rural populations—particularly in Tajikistan and Kyrgyzstan—provides essential context for understanding the observed disparities in cardiovascular disease morbidity and mortality across the region. Despite ongoing urbanization trends, the persistence of large rural communities influences access to healthcare services, disease detection, and management outcomes [42,43].

Our analysis of official national statistics revealed notable variations in CVD mortality rates both between countries and between urban and rural populations within countries. For instance, Kazakhstan demonstrated a steady decline in overall CVD mortality from 2021 to 2023, yet consistently higher mortality rates were observed in urban compared to rural populations. This urban predominance may reflect better reporting systems in cities, a higher concentration of high-risk individuals due to lifestyle factors, or disparities in healthcare utilization [25,28,43]. Conversely, the absence of detailed urban–rural disaggregated data in Kyrgyzstan, Uzbekistan, and Turkmenistan limits our ability to fully elucidate these dynamics, highlighting critical gaps in epidemiological surveillance [40,41,44]. The disproportionately high rural population in some countries likely exacerbates barriers to timely diagnosis and treatment, stemming from limited healthcare infrastructure, personnel shortages, and geographic remoteness. These systemic issues are consistent with previously documented challenges in rural health service delivery globally [45,46,47]. Consequently, mortality trends in rural areas may be underreported or underestimated, further complicating the assessment of the true CVD burden.

Considering these demographic and epidemiological realities, it becomes clear that tailored strategies for CVD prevention and management must account for the distinct needs of rural populations. Programs designed to improve early detection, control of risk factors, and access to specialized care should be prioritized in rural settings to reduce existing disparities and align with the goals of sustainable development [25,48,49].

Epidemiological Trends and Mortality Patterns

Djunusbekova et al. [26] reported a significant reduction in age-standardized CVD mortality in Kazakhstan between 2011 and 2021, including a decline in ischemic heart disease (IHD) mortality from 127.8 to 86.7 per 100,000 population. Nevertheless, mortality remains disproportionately higher among males—approximately twice that of females—and in urban settings. This gender and urban bias parallels findings in other global populations, suggesting underlying behavioral, biological, and socioeconomic determinants [50,51]. Shynar K. et al. [27] documented a substantial increase in CVD prevalence in northern Kazakhstan during the COVID-19 pandemic (2015–2020), with rates rising from 1682.0 to 4784.1 per 100,000 in urban populations and from 170.8 to 342.0 per 100,000 in rural populations. The pandemic interrupted routine care and likely intensified cardiovascular risk factors, exacerbating morbidity trends. Similar pandemic-related effects have been observed internationally [52,53,54].

Studies in Kyrgyzstan reveal a high burden of CVD risk factors in rural populations. Polupanov et al. [30] identified hypertension prevalence around 34%, obesity near 25%, and dyslipidemia exceeding 88%, with male smoking rates at 46.9%. These findings highlight critical health vulnerabilities within rural cohorts. Comparable risk factor profiles have been noted in rural Uzbekistan, where smoking and alcohol use prevalence reach 41.2% and 43.5%, respectively [32]. Mukasheva et al. [28] emphasized urban–rural disparities in Kazakhstan, observing elevated CVD incidence and mortality in urban centers, yet noting rapid increases in rural morbidity likely due to limited access to quality healthcare and preventive services. Berdesheva et al. [29] reinforced this upward trend in rural outpatient settings between 2015 and 2020.

Data from Kyrgyzstan’s Osh region, as reported by Gulamov et al. [31], indicated a rural CVD incidence of 2391.5 per 100,000 and mortality of 248.1 per 100,000 in 2021, evidencing a significant rural disease burden.

Healthcare Access and Prevention Strategies

Glushkova et al. [25] described considerable disparities in CVD prevention program availability, favoring urban areas equipped with more robust tertiary care facilities and preventive initiatives. Rural areas suffer from limited diagnostic capacity, lower screening coverage, and fewer lifestyle intervention programs, resulting in delayed diagnoses and suboptimal disease management. This rural healthcare deficit is a recurrent theme in the Central Asian epidemiological literature [55,56]. The COVID-19 pandemic further exacerbated these disparities by disrupting continuity of care, especially in rural settings, which hampered effective management of chronic cardiovascular conditions and risk factors [27,57].

Behavioral and Socioeconomic Determinants

Persistent behavioral risk factors underpin the high CVD burden. Tobacco use remains prevalent among rural men, with rates exceeding 40% in both Kyrgyzstan and Uzbekistan [27,32]. Urban populations display increasing obesity and dyslipidemia linked to sedentary lifestyles and dietary transitions associated with urbanization [25,27]. Socioeconomic status and health literacy disparities amplify these risks, particularly in rural communities with limited access to health education and preventive services [58]. Community-based, culturally adapted health promotion programs have demonstrated efficacy in mitigating risk behaviors and improving prevention uptake, underscoring the importance of localized interventions [59]. Gender-specific strategies are warranted given the higher male prevalence of smoking and hypertension [26,27]. Higher cardiovascular mortality in urban areas may also be partially driven by increased exposure to ambient air pollution, including PM_2.5_ and NO_2_, which are particularly elevated in major Central Asian cities [60]. Reductions in air pollution during COVID-19 lockdowns have been associated with a paradoxical decline in acute myocardial infarction events, as reported by Gorini et al. (2020), suggesting that environmental factors contribute meaningfully to the observed urban–rural mortality gradients [61]. Similar rural health challenges have been documented in other low- and middle-income regions, such as Sub-Saharan Africa and South Asia, where limited access to diagnostics, shortages of trained personnel, and delayed care similarly contribute to excess cardiovascular morbidity [62]. However, the Central Asian context remains distinct due to its post-Soviet healthcare legacy and differing epidemiological transition, which shape unique patterns of rural–urban disparities [63].

Research Gaps and Future Directions

The reviewed literature predominantly features cross-sectional and retrospective analyses, limiting insights into temporal dynamics and causal relationships. A paucity of longitudinal cohort studies tracking rural populations impedes our comprehensive understanding of disease progression and intervention impact. Additionally, data from Turkmenistan and Tajikistan remain scarce, hindering regional generalizability [64]. Molecular epidemiology and genetic profiling studies in Central Asian populations are virtually absent but hold potential to refine risk stratification and personalize interventions [65]. Integration of telemedicine and digital health technologies remains underutilized, despite their capacity to address rural healthcare barriers [66].

Policy Implications

The findings advocate for strengthening primary and secondary prevention programs in rural areas, including expanded hypertension screening, smoking cessation initiatives, and obesity management [25,28,29]. Investment in rural healthcare infrastructure and workforce development is essential to narrow urban–rural health disparities [25,63,67]. Incorporating pandemic preparedness into cardiovascular healthcare frameworks will ensure resilience and continuity of care during future crises [27]. Tailored health education and community engagement strategies targeting high-risk behaviors should be prioritized, with consideration of local cultural and socioeconomic contexts [27,32,68]. Finally, enhanced research capacity and regional data harmonization are imperative to inform evidence-based policy and optimize CVD prevention and control efforts in Central Asia [66,69].

## 5. Conclusions

The analysis revealed significant demographic and epidemiological features of cardiovascular diseases in Central Asian countries, with an emphasis on differences between urban and rural populations. There is a downward trend in overall CVD mortality in Kazakhstan and neighboring countries, while higher incidence and mortality rates remain in urban regions compared to rural ones. The main modifiable risk factors, including hypertension, dyslipidemia, obesity, and smoking, remain key drivers of morbidity and require priority attention in prevention programs.

The data indicate the need for a comprehensive, intersectoral approach to reducing the burden of CVD, including improving the availability and quality of healthcare in rural areas, as well as strengthening preventive strategies considering regional characteristics and the socioeconomic context. The introduction of screening and risk factor management programs aimed at vulnerable groups of the population can significantly improve the efficiency of healthcare and reduce the burden on the system.

Prospects for further research are related to an in-depth study of the impact of socioeconomic determinants, as well as an assessment of the effectiveness and adaptation of intervention programs in various demographic and geographic conditions of the region. Strengthening epidemiological monitoring and developing regional databases will contribute to more accurate analysis and timely management decisions. Thus, the results of this study are of practical value for the formation of public health policy and aim to optimize the prevention and treatment of cardiovascular diseases in Central Asian countries.

## Figures and Tables

**Table 1:** Inclusion and exclusion criteria for publication selection.

Category	Inclusion Criteria	Exclusion Criteria
Type of research	Original epidemiological studies: retrospective or prospective cohort studies, cross-sectional surveys, or comparative observational studies.	Duplicate publications presenting the same dataset without new analysis. Studies with unverifiable or unclear methodology, preventing quality assessment.
Population	Populations residing in Central Asian countries with an explicit rural–urban classification, or rural-only populations.	Studies that initially appear eligible but provide no extractable rural–urban data upon full-text review.
Outcomes	Incidence, prevalence, mortality, or DALYs for cardiovascular diseases with rural–urban stratification or rural-only reporting.	Studies with incomplete or inconsistent reporting of CVD outcomes relevant to this review.
Geographic scope	Kazakhstan, Kyrgyzstan, Uzbekistan, Tajikistan, and Turkmenistan.	Studies conducted outside the Central Asian region.
Language and access	Full-text articles available in English or Russian.	Full text unavailable after attempts to retrieve; articles lacking accessible data
Originality of data	Studies presenting original epidemiological data.	Duplicate datasets reused without new or updated results; secondary analyses lacking independent data extraction.

**Table 2:** Qualitative assessment of included studies using the Newcastle–Ottawa Scale (NOS).

ID	Author(s), Year	Selection (Max 4)	Comparability (Max 2)	Outcome (Max 3)	Total Score (Max 9)	Conclusion
1	Glushkova et al., 2023 [25]	4	2	3	9	High quality; national dataset, robust methodology, urban–rural breakdown; mortality not reported
2	Djunusbekova et al., 2023 [26]	4	2	3	9	High quality; national data, detailed statistics, no urban–rural stratification
3	Shynar K. et al., 2024 [27]	4	2	3	9	High quality; predictive modeling, urban–rural stratification, COVID-19 period coverage
4	Mukasheva et al., 2021 [28]	4	2	3	9	High quality; detailed urban–rural breakdown, incidence and mortality data
5	Berdeshova et al., 2024 [29]	3	1	2	6	Medium quality; urban–rural breakdown available, but no standardized rates per 100,000
6	Polupanov et al., 2020 [30]	3	1	2	6	Medium–high quality; rural-only focus on risk factors, no incidence/mortality data
7	Gulamov et al., 2024 [31]	4	2	3	9	High quality; detailed rural–urban dynamics, multi-year trend data, cause-specific structure
8	Salakhidinov et al., 2021 [32]	4	2	3	9	High quality; large sample size, precise diagnostic criteria, rural population only

**Table 3:** Demographic profile of Central Asian countries.

Country	Total Population	Urban Population	Rural Population
Kazakhstan [33]	20,387,811	12,899,438	7,488,373
Uzbekistan [34]	37,900,000	19,300,000	18,600,000
Kyrgyzstan [35]	7,281,800	3,073,300	4,244,500
Tajikistan [36]	10,499,000	3,028,712	7,470,288
Turkmenistan [37]	7,057,841	3,321,497	3,736,344

**Table 4:** Cardiovascular mortality statistics from national sources.

	Year	General Mortality of the Population According to ICD I00-I99 (per 100,000 People)	City	Rural
Kazakhstan [38]	2021	226.86	268.28	166.71
	2022	154.39	170.24	128.92
	2023	144.45	155.47	126.50
	2024	147.45	157.30	130.00
Kyrgyzstan [39]	2020	317.70	—	—
	2021	297.10	—	—
	2022	234.50	—	—
	2023	232.70	—	—
	2024	230.30	—	—
Tajikistan [40]	2020	205.90	224.70	199.20
	2022	142.80	171.30	131.30
	2023	147.60	180.50	134.20
Uzbekistan [40]	2020	200.40	—	—
	2021	308.50	—	—
	2022	301.10	—	—
	2023	299.60	—	—
Turkmenistan [41]	2021	552.00	—	—

**Table 5:** Summarized characteristics of the studies included in this systematic review on cardiovascular diseases in urban and rural populations of Central Asia.

ID	Author(s), Year	Country	Region(s)	Study Design	Years of Data	Population (n)	Age Group	Urban/Rural (Breakdown)	Indicators (Incidence/Prevalence/Mortality/DALY)
1	Glushkova N. et al., 2023 [25]	Kazakhstan	National, breakdown by regions (14 regions)	Content analysis of policy documents + retrospective analysis of Ministry of Health (MoH) official statistics	2006–2020 (incidence data extracted); projections to 2030	Adult population, 18+ (national data)	Adults (18+)	Yes—breakdown of urban vs. rural by incidence (AH, IHD, AMI, cerebrovascular)	Incidence (AH, IHD, AMI, cerebrovascular); projections for AMI and cerebrovascular disease; mortality not analyzed
2	Djunusbekova G., Tundybaeva M., Akhtayeva N., Kosherbayeva L., 2023 [26]	Kazakhstan	National (whole country, breakdown by gender and age)	Analysis of official statistics (Bureau of National Statistics) with AAPC calculation (Joinpoint regression)	2011–2021	Total population, 0–74 years	All age groups (main analysis 0–74)	No (aggregate)	Mortality (avoidable, preventable, treatable; age-standardized)
3	Shynar K., et. al., 2024 [27]	Kazakhstan	Northern regions (five districts, three cities, including a city of regional significance)	Descriptive research with forecasting based on official statistics	2015–2020 (with forecast until 2025)	Adult population (18+) registered at dispensaries with diagnoses I25.0–I25.9	Adults (18+)	Yes—urban vs. rural	Prevalence (CVD)
4	Mukasheva G. et al., 2021 [28]	Kazakhstan	National (14 regions)	Descriptive, MoH data	2019	—	—	Yes	Incidence and mortality (CVD, IHD, AH, AMI)
5	Berdeshova G. et al., 2024 [29]	Kazakhstan	Northern regions	Retrospective outpatient registry analysis	2015–2020	12,315 registered	All ages adult	Yes—urban–rural	Incidence trends of chronic CVD
6	Polupanov AG et al., 2020 [30]	Kyrgyzstan	Chui region (rural)	Cross-sectional epidemiology study	~2020	1330	Adults 18–65	Only rural	Prevalence of CV risk factors
7	Gulamov I. et al., 2024 [31]	Kyrgyzstan	Osh region (Alai, Chon–Alai districts)	Retrospective epidemiological analysis	2014–2021	Adults, adolescents, children < 14 (rural–urban comparison)	All age groups	Yes—rural focus	Incidence, prevalence, mortality (CVD, AH, CHD, CeVD, rheumatic heart disease)
8	Salakhidinov A., Yuldashev R.N., Kasimova N.D., 2021 [32]	Uzbekistan	Andijan region, two rural districts	Screening epidemiological study	not indicated (in context—modern period, until 2021)	17,300 adults	18+	Yes—rural population	Prevalence (smoking, alcohol, obesity, coronary heart disease by ECG, angina pectoris)

Common abbreviations used in the table include AH—arterial hypertension; IHD—ischemic heart disease; AMI—acute myocardial infarction; CVD—cardiovascular disease; CeVD—cerebrovascular disease; AAPC—average annual percent change; ECG—electrocardiogram.

**Table 6:** Summary of epidemiological indicators and risk factors reported in the included studies.

Study	Country/Region	Outcome Type	Population	Key Findings
Glushkova et al., 2023 [25]	Kazakhstan	Incidence trends	Adults 18+	↑ AH incidence in urban (462 → 1566.8/100 k) and rural; stable IHD; projected ↑ AMI and CeVD.
Dzhunusbekova et al., 2023 [26]	Kazakhstan	Mortality	National 0–74	↓ IHD mortality (127.8 → 86.7/100 k), ↓ CeVD (115.7 → 71.6), male mortality ≈ 2 × female.
Shynar K. et al., 2024 [27]	Kazakhstan	Prevalence	North Kazakhstan	Urban ↑ CVD prevalence (1682 → 4784/100 k); rural ↑ (170.8 → 342/100 k); projected further rise.
Mukasheva et al., 2021 [28]	Kazakhstan	Incidence and mortality	National	Large regional variation; highest incidence in urban Akmola and rural East Kazakhstan.
Berdesheva et al., 2024 [29]	Kazakhstan	Outpatient incidence	North Kazakhstan	Urban predominance (87.3%); rising trends in both rural and urban settings.
Polupanov et al., 2020 [30]	Kyrgyzstan	Risk factors	Rural adults	High prevalence: HTN 34%, abdominal obesity 52%, dyslipidemia 88%.
Gulamov et al., 2024 [31]	Kyrgyzstan (Osh)	Incidence and mortality	Rural population	Rural incidence 2391.5/100 k; mortality 248.1/100 k; both lower than urban Osh.
Salakhidinov et al., 2021 [32]	Uzbekistan	Risk factors	Rural adults	Smoking 41.2%; alcohol 43.5%; obesity 9.7%; angina more frequent with HTN.

**Table 7:** Key trends and data gaps identified in the selected studies.

Trend/Fact	Number of Studies Confirming	Countries/Regions	Brief Description	Areas with Data Gaps
Increase in arterial hypertension prevalence in urban and rural areas	3	Kazakhstan, Kyrgyzstan	Rising hypertension rates in urban Kazakhstan and stable/increasing trends in rural Kyrgyzstan	Turkmenistan, Tajikistan—lack of rural hypertension data
High prevalence of smoking among rural men	4	Uzbekistan, Kyrgyzstan	40–47% smoking prevalence among men in rural populations	Limited data on female smoking and age groups
Urban–rural differences in cardiovascular mortality	2	Kazakhstan, Tajikistan	Mortality rates higher in urban areas, but some regions show elevated rural mortality	Limited mortality data for Kyrgyzstan and Turkmenistan
Insufficient stratification by age and sex	5	All countries	Many studies lack detailed demographic breakdowns	Deficiency of data for targeted preventive measures
Limited data on obesity and dyslipidemia	3	Kyrgyzstan, Uzbekistan	High prevalence reported but data coverage is inconsistent	No data available for Tajikistan and Turkmenistan

## Data Availability

The original contributions presented in this study are included in this article/the Supplementary Material. Further inquiries can be directed to the corresponding author(s).

## Supplementary Materials

The following supporting information can be downloaded at: https://www.mdpi.com/article/10.3390/epidemiologia7010010/s1, Table S1: The completed PRISMA checklist. Table S2: The search strategies for database.

## References

[1] Mensah G.A., Fuster V., Murray C.J., Roth G.A., Abate Y.H., Abbasian M., Abd-Allah F., Abdollahi A., Abdollahi M., Abdulah D.M. (2023). Global Burden of Cardiovascular Diseases and Risks, 1990–2022. J. Am. Coll. Cardiol..

[2] Sethi Y., Patel N., Kaka N., Kaiwan O., Kar J., Moinuddin A., Goel A., Chopra H., Cavalu S. (2023). Precision Medicine and the future of Cardiovascular Diseases: A Clinically Oriented Comprehensive Review. J. Clin. Med..

[3] Roth G.A., Mensah G.A., Johnson C.O., Addolorato G., Ammirati E., Baddour L.M., Barengo N.C., Beaton A.Z., Benjamin E.J., Benziger C.P. (2020). Global Burden of Cardiovascular Diseases and Risk Factors, 1990–2019: Update from the GBD 2019 Study. J. Am. Coll. Cardiol..

[4] World Health Organization (2016). World Health Statistics 2016: Monitoring Health for the SDGs Sustainable Development Goals.

[5] Lindstrom M., DeCleene N., Dorsey H., Fuster V., Johnson C.O., LeGrand K.E., Mensah G.A., Razo C., Stark B., Turco J.V. (2022). Global Burden of Cardiovascular Diseases and Risks Collaboration, 1990–2021. J. Am. Coll. Cardiol..

[6] Safiri S., Karamzad N., Singh K., Carson-Chahhoud K., Adams C., Nejadghaderi S.A., Almasi-Hashiani A., Sullman M.J.M., Mansournia M.A., Bragazzi N.L. (2022). Burden of ischemic heart disease and its attributable risk factors in 204 countries and territories, 1990–2019. Eur. J. Prev. Cardiol..

[7] Di Cesare M., Perel P., Taylor S., Kabudula C., Bixby H., Gaziano T.A., McGhie D.V., Mwangi J., Pervan B., Narula J. (2024). The Heart of the World. Glob. Heart.

[8] Zhao D. (2021). Epidemiological Features of Cardiovascular Disease in Asia. JACC Asia.

[9] Dumcheva A., Nevalainen J., Laatikainen T., Nuorti P. (2024). How likely are Eastern European and central Asian countries to achieve global NCD targets: Multi-country analysis. BMC Public Health.

[10] Fonken P., Bolotskikh I., Pirnazarova G.F., Sulaimanova G., Talapbek Kyzy S., Toktogulova A. (2020). Keys to Expanding the Rural Healthcare Workforce in Kyrgyzstan. Front. Public Health.

[11] Sodiqova D., Muhsinzoda G., Dorghabekova H., Makhmudova P., Egamov F., Dastan I., Rechel B., Robinson S. (2025). Tajikistan: Health System Review. Health Syst. Transit..

[12] Supiyev A., Nurgozhin T., Zhumadilov Z., Peasey A., Hubacek J.A., Bobak M. (2017). Prevalence, awareness, treatment and control of dyslipidemia in older persons in urban and rural population in the Astana region, Kazakhstan. BMC Public Health.

[13] Goh R.S.J., Chong B., Jayabaskaran J., Jauhari S.M., Chan S.P., Kueh M.T.W., Shankar K., Li H., Chin Y.H., Kong G. (2024). The burden of cardiovascular disease in Asia from 2025 to 2050: A forecast analysis for East Asia, South Asia, South-East Asia, Central Asia, and high-income Asia Pacific regions. Lancet Reg. Health West. Pac..

[14] Collins D., Laatikainen T., Farrington J. (2020). Implementing essential interventions for cardiovascular disease risk management in primary healthcare: Lessons from Eastern Europe and Central Asia. BMJ Glob. Health.

[15] Zhang T., Li T., Jin P. (2025). Global, regional, and national burden of cardiovascular disease attributable to kidney dysfunction (1990–2021) with projections to 2050: Analysis of the 2021 Global Burden of Disease study. Ren. Fail..

[16] Righi L., Cullati S., Chopard P., Courvoisier D.S. (2022). General and Vulnerable Population’s Satisfaction with the Healthcare System in Urban and Rural Areas: Findings from the European Social Survey. Int. J. Public Health.

[17] Shaltynov A., Rocha J., Jamedinova U., Myssayev A. (2022). Assessment of primary healthcare accessibility and inequality in north-eastern Kazakhstan. Geospat. Health.

[18] Gruca T.S., Pyo T.H., Nelson G.C. (2016). Providing Cardiology Care in Rural Areas Through Visiting Consultant Clinics. J. Am. Heart Assoc..

[19] Nixon G., Davie G., Whitehead J., Miller R., de Graaf B., Liepins T., Lawrenson R., Crengle S. (2024). Rural-urban variation in the utilisation of publicly funded healthcare services: An age-stratified population-level observational study. N. Z. Med. J..

[20] Ohta R. (2025). Bridging the Digital Healthcare Gap in Rural Areas to Strengthen Communities and Enhance Care Delivery. Cureus.

[21] Faridi B., Davies S., Narendrula R., Middleton A., Atoui R., McIsaac S., Alnasser S., Lopes R.D., Henderson M., Healey J.S. (2025). Rural–urban disparities in mortality of patients with acute myocardial infarction and heart failure: A systematic review and meta-analysis. Eur. J. Prev. Cardiol..

[22] https://www.crd.york.ac.uk/PROSPERO/view/CRD420251164147.

[23] Page M.J., McKenzie J.E., Bossuyt P.M., Boutron I., Hoffmann T.C., Mulrow C.D., Shamseer L., Tetzlaff J.M., Akl E.A., Brennan S.E. (2021). The PRISMA 2020 statement: An updated guideline for reporting systematic reviews. BMJ.

[24] Wells G.A., Shea B., O’Connell D., Pereson J., Welch V., Losos M., Tugwell P. The Newcastle–Ottawa Scale (NOS) for Assessing the Quality of Nonrandomised Studies in Meta-Analyses. The Ottawa Hospital Research Institute. https://www.ohri.ca/programs/clinical_epidemiology/oxford.asp.

[25] Glushkova N., Turdaliyeva B., Kulzhanov M., Karibayeva I.K., Kamaliev M., Smailova D., Zhamakurova A., Namazbayeva Z., Mukasheva G., Kuanyshkalieva A. (2023). Examining disparities in cardiovascular disease prevention strategies and incidence rates between urban and rural populations: Insights from Kazakhstan. Sci Rep..

[26] Djunusbekova G., Tundybaeva M., Akhtayeva N., Kosherbayeva L. (2023). Recent trends in cardiovascular disease mortality in Kazakhstan. Vasc. Health Risk Manag..

[27] Shynar K., Laura S., Gulshara B., Roza S., Assel S., Maral Y. (2024). Cardiovascular diseases increased among rural and urban populations in the northern regions of the Republic of Kazakhstan during the COVID-19 period: A descriptive study with forecasting. Rev. Cardiovasc. Med..

[28] Mukasheva G., Bulegenov T., Kolyado V., Kazyeva A. (2021). Rural-urban health disparities for cardiovascular disease in the Republic of Kazakhstan. Open Access Maced. J. Med. Sci..

[29] Berdeshova G., Musina A., Orakbay L., Tolegenova A., Zhorabek S., Amanova A., Kulbayeva S. (2024). Trend of cardiovascular diseases in the northern regions of the Republic of Kazakhstan at the outpatient level. Iran. J. Public Health.

[30] Polupanov A.G., Khalmatov A., Altymysheva A., Lunegova O.S., Mirrakhimov A.E., Sabirov I.S., Kontsevaya A., Dzhumagulova A., Mirrakhimov E. (2020). The prevalence of major cardiovascular risk factors in a rural population of the Chui region of Kyrgyzstan: The Results of an epidemiological study. Anatol. J. Cardiol..

[31] Gulamov I., Abylov K., Raiimbek U.N., Satyvaldiev M., Kalmatov R. (2024). Indicators of morbidity and mortality from cardiovascular diseases of rural population in the Osh region (Kyrgyz Republic). Univ. Soc..

[32] Salakhidinov A., Yuldashev R.N., Kasimova N.D. (2021). Prevalence of risk factors for arterial hypertension among the rural population. Re-Health J..

[33] Bureau of National Statistics of the Republic of Kazakhstan *Population Statistics*. https://stat.gov.kz/ru/.

[34] State Committee of the Republic of Uzbekistan on Statistics *Demography Indicators*. https://stat.uz/ru/ofitsialnaya-statistika/demography.

[35] National Statistical Committee of the Kyrgyz Republic *Population Statistics*. https://stat.gov.kg/ru/statistics/naselenie/.

[36] Agency on Statistics Under the President of the Republic of Tajikistan *Official Statistical Data*. https://www.stat.tj/.

[37] State Statistics Committee of Turkmenistan *Official Statistical Information*. https://www.stat.gov.tm/ru.

[38] National Research Center for Health Development (NRCHD) *Health of the Population of the Republic of Kazakhstan and Activities of Healthcare Organizations, 2021–2024*. https://nrchd.kz/.

[39] National Statistical Committee of the Kyrgyz Republic *Healthcare Statistics*. https://stat.gov.kg/ru/statistics/zdravoohranenie/.

[40] State Committee of the Republic of Uzbekistan on Statistics *National Indicators for Sustainable Development Goals*. https://nsdg.stat.uz/goal/6.

[41] World Heart Federation *World Heart Observatory: Turkmenistan Country Profile*. https://world-heart-federation.org/world-heart-observatory/countries/turkmenistan/.

[42] Münzel T., Hahad O., Sørensen M., Lelieveld J., Duerr G.D., Nieuwenhuijsen M., Daiber A. (2022). Environmental risk factors and cardiovascular diseases: A comprehensive expert review. Cardiovasc. Res..

[43] Mirrakhimov E., Zakirov U., Abilova S., Asanbaev A., Bektasheva E., Alibaeva N., Neronova K., Kerimkulova A., Altymysheva A., Wang W. (2022). May Measurement Month 2019: Analysis of Blood Pressure Screening in Bishkek, Kyrgyzstan. Eur. Heart J. Suppl..

[44] Yuan J.J., Lu Y.L., Ferrier R.C., Liu Z.Y., Su H.Q., Meng J., Song S., Jenkins A. (2018). Urbanization, rural development and environmental health in China. Environ. Dev..

[45] Kumar P., Lionis C., Andoko D., Rahman Z., Anastasaki M., Awankem B. (2025). Evaluation of Diagnostic Services in Rural and Remote Areas: Bottlenecks, Success Stories, and Solutions. J. Surg. Spec. Rural Pract..

[46] Afni N. (2023). Disparities in Healthcare Access: Addressing Systemic Barriers in Urban and Rural Communities. J. Health Lit. Qual. Res..

[47] Maganty A., Byrnes M.E., Hamm M., Wasilko R., Sabik L.M., Davies B.J., Jacobs B.L. (2023). Barriers to rural health care from the provider perspective. Rural Remote Health.

[48] Palozzi G., Schettini I., Chirico A. (2020). Enhancing the Sustainable Goal of Access to Healthcare: Findings from a Literature Review on Telemedicine Employment in Rural Areas. Sustainability.

[49] Buse K., Hawkes S. (2015). Health in the sustainable development goals: Ready for a paradigm shift?. Glob. Health.

[50] Vervoort D., Wang R., Li G., Filbey L., Maduka O., Brewer L.C., Mamas M.A., Bahit M.C., Ahmed S.B., Van Spall H.G.C. (2024). Addressing the global burden of cardiovascular disease in women: JACC state-of-the-art review. J. Am. Coll. Cardiol..

[51] Connelly P.J., Azizi Z., Alipour P., Delles C., Pilote L., Raparelli V. (2021). The importance of gender to understand sex differences in cardiovascular disease. Can. J. Cardiol..

[52] Janus S.E., Makhlouf M., Chahine N., Motairek I., Al-Kindi S.G. (2022). Examining disparities and excess cardiovascular mortality before and during the COVID-19 pandemic. Mayo Clin. Proc..

[53] Adair T. (2023). Premature cardiovascular disease mortality with overweight and obesity as a risk factor: Estimating excess mortality in the United States during the COVID-19 pandemic. Int. J. Obes..

[54] Song S., Guo C., Wu R., Zhao H., Li Q., Dou J.H., Guo F.S., Wei J. (2024). Impact of the COVID-19 pandemic on cardiovascular mortality and contrast analysis within subgroups. Front. Cardiovasc. Med..

[55] Rechel B., Ahmedov M., Akkazieva B., Katsaga A., Khodjamurodov G., McKee M. (2012). Lessons from two decades of health reform in Central Asia. Health Policy Plan..

[56] Aringazina A., Kuandikov T., Arkhipov V. (2018). Burden of the Cardiovascular Diseases in Central Asia. Cent. Asian J. Glob. Health.

[57] Russo R.G., Li Y., Ðoàn L.N., Ali S.H., Siscovick D., Kwon S.C., Yi S.S. (2021). COVID-19, social determinants of health, and opportunities for preventing cardiovascular disease: A conceptual framework. J. Am. Heart Assoc..

[58] Wang W., Zhang Y., Lin B., Mei Y., Ping Z., Zhang Z. (2020). The Urban-Rural Disparity in the Status and Risk Factors of Health Literacy: A Cross-Sectional Survey in Central China. Int. J. Environ. Res. Public Health.

[59] Shafique S., Bhattacharyya D.S., Nowrin I., Sultana F., Islam R., Dutta G.K., del Barrio M.O., Reidpath D.D. (2024). Effective community-based interventions to prevent and control infectious diseases in urban informal settlements in low- and middle-income countries: A systematic review. Syst. Rev..

[60] Al-Kindi S.G., Brook R.D., Biswal S., Rajagopalan S. (2020). Environmental determinants of cardiovascular disease: Lessons learned from air pollution. Nat. Rev. Cardiol..

[61] Gorini F., Chatzianagnostou K., Mazzone A., Bustaffa E., Esposito A., Berti S., Bianchi F., Vassalle C. (2020). “Acute Myocardial Infarction in the Time of COVID-19”: A Review of Biological, Environmental, and Psychosocial Contributors. Int. J. Environ. Res. Public Health.

[62] Moiynbayeva S., Akhmetov V., Narymbayeva N., Shaikova K., Makhanbetkulova D., Bapayeva M., Abdirova T., Popova T., Karibayeva I. (2024). Health policy implications for cardiovascular disease, type 2 diabetes mellitus, and stroke in Central Asia: A decadal forecast of their impact on women of reproductive age. Front. Public Health.

[63] Jain N., Nagaich U., Pandey M., Chellappan D.K., Dua K. (2022). Predictive genomic tools in disease stratification and targeted prevention: A recent update in personalized therapy advancements. EPMA J..

[64] Sommer D., Wilhelm S., Ahrens D., Wahl F. Implementing an Intersectoral Telemedicine Network in Rural Areas: Evaluation from the Point of View of Telemedicine Users. Proceedings of the International Conference on Information and Communication Technologies for Ageing Well and e-Health.

[65] Franco C.M., Lima J.G., Giovanella L. (2021). Primary healthcare in rural areas: Access, organization, and health workforce in an integrative literature review. Cad. Saúde Pública.

[66] Reilly M. (2021). Health Disparities and Access to Healthcare in Rural vs. Urban Areas. Theory Action.

[67] Boyd R.C., Castro F.G., Finigan-Carr N., Okamoto S.K., Barlow A., Kim B.-K.E., Lambert S., Lloyd J., Zhang X., Barksdale C.L. (2023). Strategic Directions in Preventive Intervention Research to Advance Health Equity. Prev. Sci..

[68] Yi S., Yam E.L.Y., Cheruvettolil K., Linos E., Gupta A., Palaniappan L., Rajeshuni N., Vaska K.G., Schulman K., Eggleston K.N. (2024). Perspectives of digital health innovations in low-and middle-income health care systems from South and Southeast Asia. J. Med. Internet Res..

[69] Zou K.H., Li J.Z., Salem L.A., Imperato J., Edwards J., Ray A. (2021). Harnessing real-world evidence to reduce the burden of noncommunicable disease: Health information technology and innovation to generate insights. Health Serv. Outcomes Res. Methodol..

